# A novel Coltivirus-related virus isolated from free-tailed bats from Côte d’Ivoire is able to infect human cells in vitro

**DOI:** 10.1186/s12985-017-0843-0

**Published:** 2017-09-18

**Authors:** Sabrina Weiss, Piotr Wojtek Dabrowski, Andreas Kurth, Siv Aina J. Leendertz, Fabian H. Leendertz

**Affiliations:** 1 Robert Koch-Institut, Epidemiology of Highly Pathogenic Microorganisms (P3), Seestrasse 10, 13353 Berlin, Germany; 2 Robert Koch-Institut, Methodology and Research Infrastructure 1 - Bioinformatics, Seestraße 10, 13353 Berlin, Germany; 3 Robert Koch-Institut, Centre for Biological Threats and Special Pathogens 1 (ZBS1), Seestraße 10, 13353 Berlin, Germany; 4 Robert Koch-Institut, Biosafety Level 4-Laboratory (ZBS5), Seestrasse 10, 13353 Berlin, Germany; 5 Current Address: Charité – Universitätsmedizin Berlin, Institute of Virology, Charitéplatz 1, 10117 Berlin, Germany

**Keywords:** *Reoviridae*, Chiroptera, Bats, C*oltivirus*, *Chaereophon aloysiisabaudiae*, Colorado tick fever, *Spinareovirinae*

## Abstract

**Background:**

Zoonotic transmission events play a major role in the emergence of novel diseases. While such events are virtually impossible to predict, wildlife screening for potential emerging pathogens can be a first step. Driven by recent disease epidemics like severe acute respiratory syndrome (SARS), Middle East respiratory syndrome (MERS), and Ebola, bats have gained special interest as reservoirs of emerging viruses.

**Methods:**

As part of a bigger study investigating pathogens in African bats we screened animals for the presence of known and unknown viruses.

**Results:**

We isolated and characterised a novel reovirus from blood of free-tailed bats (*Chaereophon aloysiisabaudiae*) captured in 2006 in Côte d’Ivoire. The virus showed closest relationship with two human pathogenic viruses, Colorado tick fever virus and Eyach virus, and was able to infect various human cell lines in vitro.

**Conclusion:**

The study shows the presence of a coltivirus-related virus in bats from Sub-Sahara Africa. Serological studies could help to assess its impact on humans or wildlife health.

## Background

Zoonotic transmission events play a major role in the emergence of novel diseases. While it is difficult to predict the emergence of zoonotic pathogens, wildlife screening of healthy animals for novel pathogens and evaluation of their capacity to induce disease can be a first step [1]. Among mammals, bats have gained special interest as potential reservoirs of emerging viruses such as severe acute respiratory syndrome (SARS), Middle East respiratory syndrome (MERS), and Ebola [2, 3]. A number of reoviruses (family *Reoviridae*; respiratory, enteric, orphan virus) have been described in bats in Europe and Asia, all of which belong to the genus *Orthoreovirus* in the *Spinareovirinae* subfamily (e.g. [4–9, 10, 11]). Some of these have also been isolated from humans suffering from acute respiratory or gastrointestinal illness and likely originated from bats [4–6, 12]. As part of a bigger study [13, 14] we investigated bats from Sub-Sahara Africa for the presence of pathogens with zoonotic potential.

The family *Reoviridae* harbors viruses with a segmented double-stranded RNA genome. It is currently divided into two subfamilies; the *Sedoreovirinae* comprising six, and the *Spinareovirinae* comprising nine genera [15]. The genus *Coltivirus* in the latter subfamily is comprised of two species only: Colorado tick fever virus (CTFV) and Eyach virus (EYAV). CTFV is the etiologic agent of a febrile human disease, Colorado tick fever, occurring in the Rocky Mountains in the Western United States and Canada [16–18]. It is rarely fatal but can cause severe complications like encephalitis, haemorrhage, or pericarditis, especially in children [19]. The related EYAV was isolated in Germany in 1976 and has been associated with human neurological disease by serological evidence [20, 21]. Animal reservoirs of coltiviruses are small mammals like rodents and lagomorphs and transmission to humans occurs by ticks of the family *Ixodida*e [18, 22, 23]. We describe a novel reovirus, designated Taï Forest reovirus (TFRV), which is closely related to coltiviruses, a genus not described in bats before, isolated from blood of African free-tailed bats (*Chaereophon aloysiisabaudiae*).

## Methods

### Sampling and virus isolation

Bats were captured with mist nets in November and December 2006 in the Taï National Park in Côte d’Ivoire. Blood was taken from the wing vein and mixed with EDTA to prevent coagulation. Animals were released immediately after the procedure. All samples were stored in liquid nitrogen, transported on dry ice and transferred to −80 °C at the Robert Koch-Institut in Berlin for long-time storage. Primary virus isolation was achieved on VeroE6 cells. For inoculation 50 μl of blood from each of three individuals *Ch. aloysiisabaudiae* was pooled. Cells were grown to 70–80% confluence in 25 cm^2^ cell culture flasks. Prior to inoculation cells were washed with phosphate buffered saline (PBS) followed by medium without supplements. Cells were inoculated with blood diluted in PBS to a final volume of 2 ml. After 1–2 h 3 ml of medium including 2% Fetal Calf Serum, 1% L-glutamine, 1% penicillin/streptomycin was added and cells were incubated at 37 °C with 5% CO_2_. After 24 h cells were washed with PBS and medium was replaced. Cells were then incubated for 7 days and monitored daily microscopically for the presence of a cytopathic effect (CPE). After 7 days 300 μl supernatant was used to infect fresh cells. A CPE was observed at day five in the second passage. To prepare virus stocks virus was grown in a 175 cm^2^ flask and virus particles isolated by ultracentrifugation through a 36% sucrose solution for 4 h at 28,000 rpm at 4 °C in a Beckmann ultracentrifuge (rotor SW 32 Ti, Beckman Coulter).

### Virus visualisation and sequencing

For visualization of viral particles cells grown in a 25 cm^2^ flask were fixed with glutaraldehyde (final concentration 2.5%) over night at 4 °C as soon as a CPE became visible. Photographic documentation of samples was performed on a FEI Tecnai G2 transmission electron microscope (TEM).

Viral RNA was isolated using the QIAamp Viral RNA Mini Kit (Qiagen) without carrier RNA and initial sequence information was generated by Particle-associated Nucleic Acid PCR [24] and subsequent cloning and Sanger sequencing of the amplicons. Of the resulting sequences nine contigs and three individual reads showed homologies with two viruses, CTFV and EYAV, when compared to GenBank entries. The CTFV genome was used as reference genome to map contigs of the novel virus so as to get a possible arrangement, based on which sequence gaps were closed with primers directed outwards from known fragments (described in detail in [25]). Segment ends of three identified segments (S1, S2, S9) were obtained by rapid amplification of cDNA ends (Thermo Fisher). In parallel, RNA was applied to 454 next generation sequencing (Roche), resulting in a total of 222,953 reads. Reads were trimmed using Trimmomatic version 0.33 [26] and all trimmed reads shorter than 250 bases were discarded. The resulting 135,911 reads were separated into pathogen and host reads using RAMBO-K version 1.2 [27] (background reference: Chlorocebus sabaeus, GCF_000409795.2; foreground references: Eyach virus, NC_003696-NC_003707, and Colorado tick fever virus, NC_004180-NC_004191) using a k-mer length of 4 and a cutoff score of −2.5, yielding 71,962 potential foreground reads. These reads were assembled together with the available Sanger sequences using SPAdes version 3.8.1 [28]. Of the 250 contigs reconstructed by SPAdes, only 38 were longer than 300 bases. BLASTX of these 38 contigs was performed against a custom database of the Eyach virus and Colorado tick fever virus genomes using Geneious Pro version 10 [29], with 10 contigs showing significant similarity to 9 known segments. In an iterative process, all reads were mapped back to the contigs, and the mapping reads were re-assembled, until no new unambiguously mapping reads could be identified and contigs could not be extended anymore. Finally, a last round of mapping all reads to all contigs was performed for error correction.

### Sequence and phylogenetic analysis

To identify putative proteins encoded by the different segments possible open reading frames (ORFs) were identified using the ORF finder in Geneious Pro. Putative gene functions were predicted based on homology to CTFV and the according UniProtKB entries [30].

For phylogenetic inference sequences of RNA dependent RNA polymerase (RdRp) genes from representative viruses across different genera within the *Spinareoviridae* were downloaded and aligned on protein level using the muscle algorithm [31] as implemented in SeaView version 4.6.1 [32]. To increase alignment quality conserved blocks were selected using the Gblocks server [33], which resulted in an alignment of 263 amino acids (aa). Maximum likelihood (ML) tree was achieved using the PhyML 3.0 server [34] using the RtREV +G + F model as determined by Smart Model Selection as implemented on the website. Sequences of available putative RNA-methyltransferase proteins from reoviruses were used for phylogenetic inference using the same pathway as described above. The resulting alignment consisted of 667 aa and the model used for phylogenetic inference was LG + G + F.

## Results

**Fig. 1:**
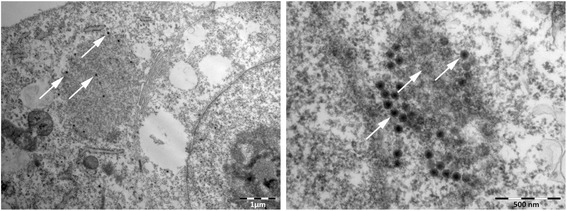
Ultra-thin sections of Vero cells infected with Taï Forest reovirus (TFRV). Cells were fixed at 3 days post infection. Pictures show reovirus-characteristic particles (white arrows) with typical inner and outer icosahedral capsids of approximately 70 nm in diameter

Assembled sequences of the novel virus showed homology to two viruses exclusively, CTFV and EYAV, which together form the genus *Coltivirus*. This was confirmed by TEM pictures showing particles of approximately 70 nm in diameter and typical inner and outer icosahedral capsids, which are characteristic for reoviruses (Fig 1).

Coltiviruses have double stranded RNA genomes comprised of 12 genomic segments with sizes ranging from 675 to 4350 nucleotides (nt). Using Sanger and 454 sequencing methodology nine segments of the novel virus could be assembled. Segments 1, 2, and 9 were completely assembled whereas for segments 3, 5, 8, 10, and 11 ends are missing. For segment 4 two long contigs were assembled but not combined to one long contig. No sequence information was identified for segments 6, 7 and 12. Table 1 provides comparison between identified segments those of CTFV and EYAV. Table 1 provides an overview about similarities between the segments and CTFV and EYAV segments. The novel virus was tentatively named Taï Forest Reovirus (TFRV), based on the origin of its host.

**Table 1:** Comparison of segments between Taï Forest reovirus (TFRV), Colorado tick fever virus (CTFV) and Eyach virus (EYAV)

	Segment length/ ORF length [nt](start nt position)			% identity at aa level (% coverage)		Putative gene function	UniprotKB (% identity^c^, % coverage)
Seg	TFRV	CTFV	EYAV	CTFV	EYAV		
S1	4356/4305 (12)	4350/4308 (14)	4349/4308 (13)	56.7 (98.6)	56.8 (98.6)	RNA-dependent RNA polymerase	Q9DSQ0 (56.8, 99.8)﻿﻿﻿
S2	3987/3903 (35)	3909/3630 (46)	3934/3828 (45)	39.1 (82.7)	38.3 (91.9)﻿﻿	Putative RNA-methyltransferase	Q9ENL4 (39.4, 84.5)﻿
S3	3513^a^	3586/3549 (12)﻿	3585/3549 (11)	28.9 (98.6)	29.1 (95.7)	Membrane-spanning protein	Q9ENL3 (28.9, 99.7)
S4–1	1794	3157/ 3084	3156/3084	41.0 (29.6)^b^	32.1 (99.2)^b^	Uncharacterised protein VP4	Q9ENL2 (43.0, 29.7)
S4–2	1398	3157/3084	3156/3084	46.7 (72.1)^b^	47.3 (72.1)^b^	Uncharacterised protein VP4	Q9ENL2 (46.7, 72.1)
S5	3041^a^/2577	2432/2256	2398/2256	30.1 (41.8)	29.7 (45.1)	Uncharacterised protein VP5	Q9ENL1 (31.1, 41.8)
S8	2176^a^	2029/1983	2028/1983	35.3 (25.9)	28.8 (53.4)	Uncharacterised protein VP8	Q9ENK8 (35.3, 26.2)
S9	1857/VP9: 1008, VP9': 1794 (33)	1884/VP9: 1014, VP9': 1809 (41)	1884/VP9: 1014, VP9': 1809 (41)	P9: 37.2 (99.7), VP9': 37.3 (94.4)	VP9: 36.3 (99.7), VP9': 36.1 (94.4)﻿﻿	Structural protein VP9, Non-structural protein VP9'	VP9: O93214 (37.2, 100), VP9': O55265 (37.7, 97.7)
S10	1845^a^	1880/1818	1879/1818	39.5 (99.7)	39.0 (99.7)	Microtubule-associated protein VP10	Q9ENK7 (39.5, 99.8)﻿
S11	971^a^/915	998/759	1002/927	26.5 (89.9)	25.3 (71.1)	Uncharacterised protein VP11	Q96713 (25.3, 75.4)

Putative protein functions are allocated based on known proteins according to UniProt database

^a^Segment ends missing. ^b^Based on partial sequences. ^c^Identity to the first Swiss-Prot reviewed entry. *Seg* Segment, *VP* virus protein

With few exceptions reovirus segments commonly contain one ORF coding for a single protein, flanked by non-coding regions and conserved terminal sequence motifs. Identified segments of TFRV are monocistronic with the exception of one segment, which putatively contains two overlapping ORFs, mediated through an Opal stop codon, followed by a cytosine residue. This construction allows for two proteins of different lengths, but beginning with the same start codon, a shorter viral protein 9 (VP9) and a longer VP9’ protein [17, 18, 35]. Segment lengths and putative start codons in comparison to CTFV and EYAV are listed in Table 1. Terminal sequence motifs identified in TFRV are A$$ \raisebox{1ex}{$\boldsymbol{A}$}\!\left/ \!\raisebox{-1ex}{$\boldsymbol{U}$}\right.\raisebox{1ex}{$\boldsymbol{A}$}\!\left/ \!\raisebox{-1ex}{$\boldsymbol{U}$}\right.\raisebox{1ex}{$\boldsymbol{A}$}\!\left/ \!\raisebox{-1ex}{$\boldsymbol{U}$}\right.\raisebox{1ex}{$\boldsymbol{A}$}\!\left/ \!\raisebox{-1ex}{$\boldsymbol{U}$}\right. $$UG$$ \raisebox{1ex}{$\boldsymbol{A}$}\!\left/ \!\raisebox{-1ex}{$\boldsymbol{U}$}\right.\raisebox{1ex}{$\boldsymbol{A}$}\!\left/ \!\raisebox{-1ex}{$\boldsymbol{U}$}\right. $$ on the 5’end and UGCAGU on the 3’end.

Virus protein (VP) 1 of TFRV contains RNA polymerase domains (UniProt Q9DSQ0) and putatively encodes for a RNA dependent RNA polymerase. This is supported by the presence of two functional motifs that are present in all reovirus RdRp genes [18]: motif SG (positions 754–755 for CTFV and EYAV, positions 753–754 for TFRV) and motif GDD (positions 816–818 for CTFV and EYAV, 814–816 for TFRV). VP2 on segment 2 might code for a RNA methyltransferase according to UniProt (Q9ENL4), VP3 on segment 3 for a membrane protein (UniProt Q9ENL3), the ORFs VP9 and VP9’ on S9 for a structural and non-structural protein (UniProt O93214 and O55265), respectively, and VP10 for a microtubule-associated protein (UniProt Q9ENK7).

Based on sequence information of segment 1, a quantitative real-time PCR (qPCR) assay was designed to determine the viral load in the original samples and to screen all bat blood samples available from Taï National Park. In total, 264 samples from 11 bat genera were tested. TFRV was only detected in samples from three *Chaerephon* bats that were used for primary inoculation of Vero cells. Virus loads of the three samples were 7.6 × 10^3^, 2.6 × 10^4^, and 2.1 × 10^5^ genome equivalents per ml.

**Fig. 2:**
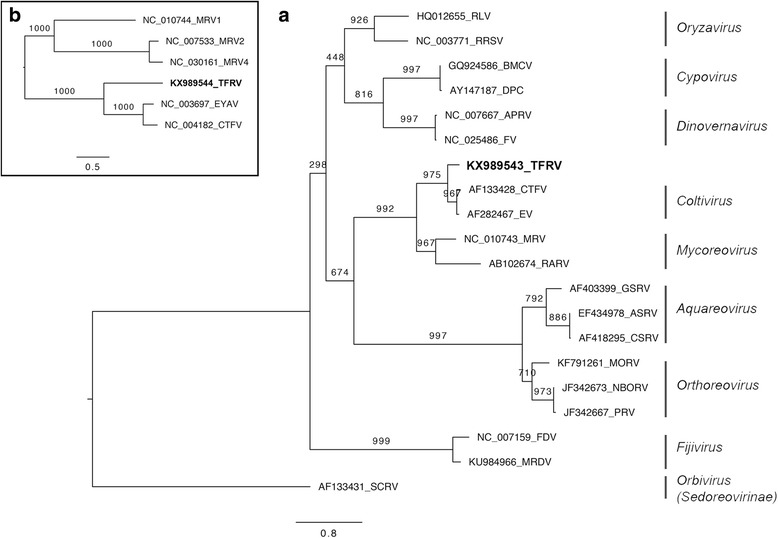
Phylogenetic trees. **a** Maximum-likelihood (ML) tree based on deduced amino acid (aa) sequences of the RdRp genes (263 aa) from selected representatives within the subfamily *Spinareoviridae*. St Croix river virus (SCRV) belongs to the subfamily *Sedoreovirinae* and was included as outgroup. **b** ML tree based on deduced aa sequences (665 aa) of available putative RNA-methyltransferase genes of members of the subfamily *Spinareoviridae*. Scale bars indicates amino acid substitutions per site. Branch values represent bootstrap values obtained from 1000 bootstraps. APRV = Aedes pseudoscutellaris reovirus, ASRV = Atlantic salmon reovirus, BMCV = Bombyx mori cypovirus 1, CSRV = Chum salmon reovirus, CTFV = Colorado tick fever virus, DPC = Dendrolimus punctatus cypovirus 1, EYAV = Eyach virus, FDV = Fiji disease virus, FV = Fako virus strain CSW77, GSRV = Golden shiner reovirus, MORV = Mammalian orthoreovirus, MRDV = Maize rough dwarf virus, MRV = Mycoreovirus, NBORV = Nelson Bay orthoreovirus, PRV = Pulau reovirus, RARV = Rosellinia anti-rot virus, RLV = Rapsberry latent virus, RRSV = Rice ragged stunt virus, TFRV = Taï Forest reovirus

The RdRp gene encoded on segment 1 and the putative RNA-methyltransferase genes encoded on segment 2 were used for phylogenetic analysis, which confirm the relationship between TFRV and Coltiviruses (Fig 2).

**Fig. 3:**
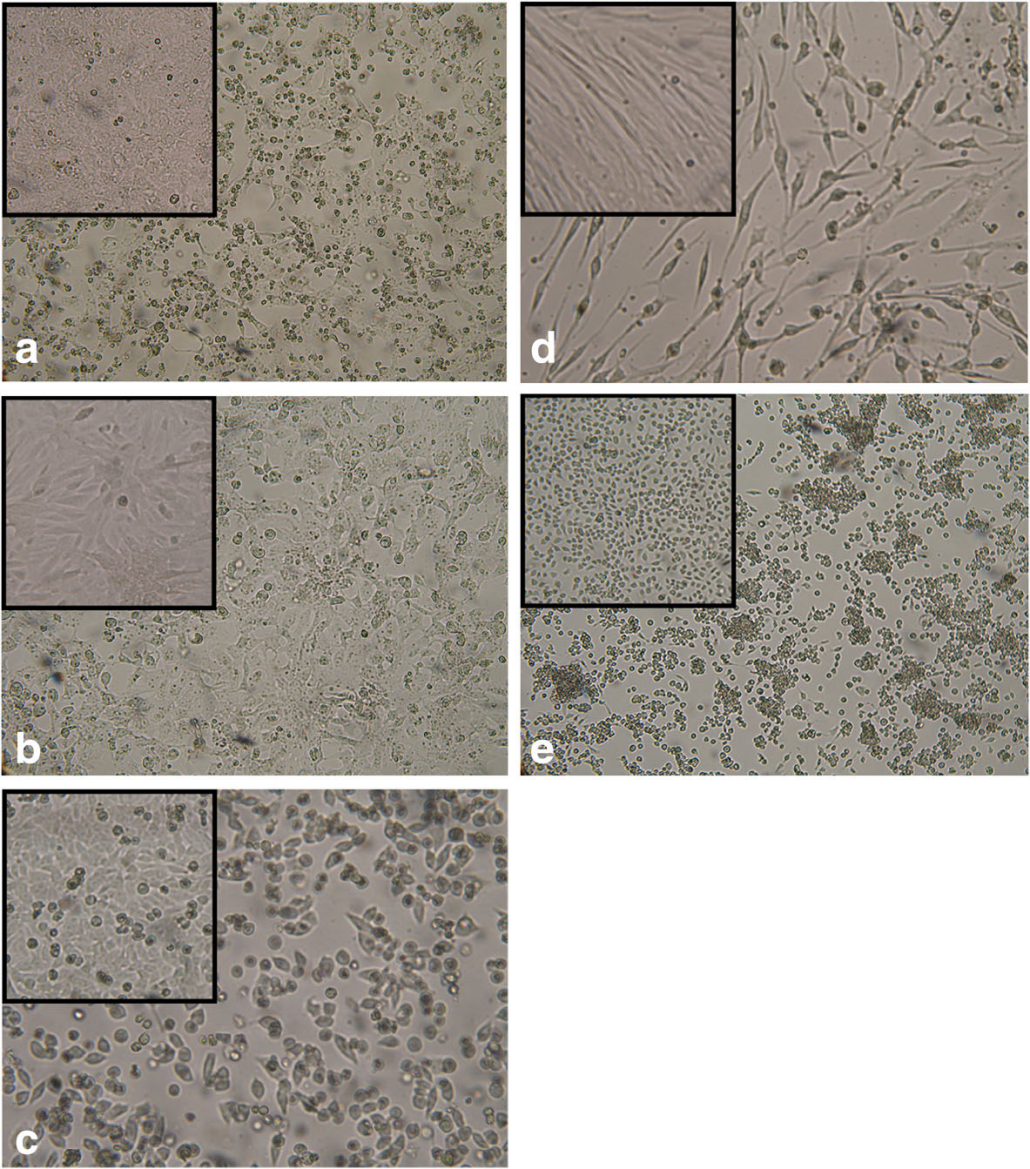
Cells infected with TFRV and uninfected control cells (upper left corner of each photo). p, passage; dpi, days post infection. **a** VeroE6, p5, 5dpi; **b** R05T, p5, 4dpi; **c** Hep2, p12, 2dpi; **d** MRC-5, p12, 3dpi; **e** C6/36, p4, 14dpi

To estimate the possible host range of TFRV it was used for inoculation of various cell lines. The virus was able to induce a CPE on C6/36 insect cells and on various mammalian cell lines (Fig 3): Primate kidney cells (VeroE6), a fruit bat cell line originating from *Rousettus aegyptiacus* (R05T), and two human cell lines, lung fibroblasts (MRC-5) and liver cells (Hep2).

## Discussion

We have isolated a novel reovirus from blood of free-tailed bats from Côte d’Ivoire that clusters together with coltiviruses in phylogenetic analysis. The isolation of TFRV from blood is in line with the observed relationship to coltiviruses. CTFV, the type species of coltiviruses, replicates in erythropoietic cells and produced virus is maintained in red blood cells [23]. The level of aa identity between homologous proteins of different strains within various genera across the *Reoviridae* family is between 20%–45% [18]. 57% aa identity of the RdRp with both, CTFV and EYAV, suggesting that TFRV belongs to the same genus, namely *Coltivirus*. For those segments identified, comparisons between TFRV and coltiviruses show identities ranging from 27% to 57%. Between homologous segments of CTFV and EYAV identities vary between 69% and 75%, exceptions being segments 6 and 7, where identities are 40% and 43% only. Assuming similarities between TFRV and CTFV or EYAV are even lower, this might explain difficulties to identify, or accurately annotate, contigs belonging to segments 6 and 7.

Coltiviruses are known to have a large host range in vivo, infecting ticks as well as a variety of mammals [18], so the host range observed in vitro for TFRV is not surprising. However, the induction of a CPE on a wide range of mammalian cells, including human cells, together with its close phylogenetic relationship to human pathogenic viruses, allows for speculation about the ability of TFRV to infect humans. Febrile symptoms, as caused by CTFV, are common in tropical regions and can account for a number of different diseases. In Africa, especially in remote areas where *Ch. aloysiisabaudiae* is found, health care systems are often not functioning optimally and most of the time no laboratory diagnostic test is performed. Hence if TFRV would cause a disease similar to CTF in humans, it would most likely remain unidentified.

Based on present data we cannot infer whether *Ch. aloysiisabaudiae* is an accidental host or represents the true reservoir of TFRV. No TFRV was detected in any other bat species, including one individual of the related species *Ch. russata*. *Ch. aloysiisabaudiae* is widely distributed across the sub-Saharan Africa. Besides the forest zones in Côte d’Ivoire and western Ghana, it is also present in Central Africa along a stretch from Cameroon to South Sudan and Uganda. Since populations are spatially separated it would be of interest to look for TFRV in central African bats to further explore its epidemiology. The screening of ticks could also be of interest. Ixodid ticks are the vector for infecting humans with CTFV and EYAV and are also present in Africa [36]. The close relationship of the viruses and the observation that TFRV infects insect cell lines in vitro allows for the possibility that it also requires ticks or another vector to spread. Despite the fact that this would limit the possibility for transmission to a region with a geographical overlap of bats, vector, and humans, the presence of the virus in blood raises concern about transmission by blood-feeding vectors. TFRV is able to infect cells from various mammals in vitro and it might be that other mammals or vectors also play a role in the virus’ ecology. Development of a serological test to screen animals and humans from the Taï region for antibodies against TFRV would help to shed light on the potential transmission of this novel reovirus in the Taï Forest Region and along the distribution of *Ch. aloysiisabaudiae.*

## Conclusion

We described a putative novel member of the *Coltivirus* family. To our knowledge, TFRV represents the first coltivirus isolated from bats and on the African continent. With respect to the pathogenic potential of coltiviruses further screening of wildlife and possible vectors could help to learn about the epidemiology of the virus and to assess its potential impact on human and animal health.

## Abbreviations

*Ch.*: *Chaereophon*
CPE: Cytopathic effect
CTRV: Colorado Tick Fever Virus
EYAV: Eyach Virus
MERS: Middle East respiratory syndrome
SARS: Severe acute respiratory syndrome
TFRV: Taï Forest Reovirus
VP: Virus protein
